# ADAM23 is a common risk gene for canine idiopathic epilepsy

**DOI:** 10.1186/s12863-017-0478-6

**Published:** 2017-01-31

**Authors:** Lotta L. E. Koskinen, Eija H. Seppälä, Jutta Weissl, Tarja S. Jokinen, Ranno Viitmaa, Reetta L. Hänninen, Pascale Quignon, Andrea Fischer, Catherine André, Hannes Lohi

**Affiliations:** 1 Research Programs Unit, Molecular Neurology, University of Helsinki, Helsinki, Finland; 2 Department of Veterinary Biosciences and Department of Medical Genetics, University of Helsinki, Helsinki, Finland; 3 Folkhälsan Institute of Genetics, Helsinki, Finland; 4 Centre for Clinical Veterinary Medicine, Ludwig-Maximilians-Universität München, Munich, Germany; 5 Department of Clinical Veterinary Sciences, University of Helsinki, Helsinki, Finland; 6 CNRS, UMR 6290, Institut de Génétique et Développement de Rennes, Rennes, France; 7 Université Rennes 1, UEB, Biosit, Faculté de Médecine, Rennes, France

**Keywords:** Dog, *Canis familiaris*, Epilepsy, Idiopathic epilepsy, *ADAM23*, GWA, Association

## Abstract

**Background:**

Idiopathic or genetic adult-onset epilepsy is a common neurological disorder in domestic dogs. Genetic association has been reported only with *ADAM23* on CFA 37 in few breeds. To identify novel epilepsy genes, we performed genome-wide association (GWA) analyses in four new breeds, and investigated the association of the previously reported *ADAM23* haplotype with the epilepsy phenotype in eight breeds.

**Results:**

GWA analysis did not reveal new epilepsy loci. *ADAM23* association (*p* < 0.05) was identified in five breeds. Combined analysis of all eight breeds showed significant association (*p* = 4.6e^−6^, OR 1.9).

**Conclusions:**

Our results further support the role of *ADAM23* in multiple breeds as a common risk gene for epilepsy with low penetrance. The lack of findings in the GWA analyses points towards inefficient capture of genetic variation by the current SNP arrays, causal variant(s) with low penetrance and possible phenocopies. Future work will include studies on ADAM23 function and expression in canine neurons, as well as whole-genome sequencing in order to identify additional IE genes.

**Electronic supplementary material:**

The online version of this article (doi:10.1186/s12863-017-0478-6) contains supplementary material, which is available to authorized users.

## Background

Idiopathic, or primarily genetic, epilepsy (IE) is the most common neurological disease in dogs with an overall prevalence of approximately 0.62% [1]. It is common across many breeds, but some breeds have higher disease prevalence, which indicates increased genetic predisposition [1–7]. Although pedigree analysis has clearly suggested strong genetic influence, the identification of risk genes has been challenging [8].

To date, two genes underlying canine IE have been identified: *LGI2* on chromosome 3 for benign juvenile epilepsy in the Lagotto Romagnolo breed [9], and *ADAM23* on chromosome 37 for focal and generalized adult-onset epilepsy in the Belgian Shepherd breed [10]. According to our recent study employing high-density genome-wide association (GWA) analysis and targeted next generation sequencing in large sample cohorts, *ADAM23* harbors a common risk haplotype for IE in Belgian Shepherd, Finnish Spitz, Schipperke and Beagle breeds [11]. The risk haplotype spans exons 5 and 11 of the *ADAM23* gene and presented with odds ratios of 3.3–12.0 depending on the breed. The results suggest that *ADAM23* is a common but a low penetrance risk gene for IE in several breeds, but other yet unknown factors may contribute to the disease risk. ADAM23 (ADAM23 metallopeptidase domain 23) is a membrane-anchored protein expressed in several tissues and involved in cell adhesion and central nervous system development. It interacts with LGI1, LGI2 and ADAM22 in synaptic transmission [12]. LGI1, LGI2 and ADAM22 have previously been implicated in epilepsy [9, 13, 14].

In this study, we aimed to identify new IE loci by GWA analyses in four breeds: Finnish Lapphund, Kromfohrländer, Miniature Pinscher and Pyrenean Shepherd. Furthermore, we investigated the role of *ADAM23* in an extended epilepsy collection of altogether eight dog breeds with IE: Australian Shepherd, Irish Setter, Labrador Retriever and Whippet, and the above mentioned breeds.

## Methods

In order to identify genes underlying canine epilepsy, we investigated the characteristics of IE in the studied breeds, collected DNA samples from cases and controls with IE and performed GWA analyses in four dog breeds. We also studied the association of the *ADAM23* gene with IE in eight dog breeds.

### Sample collection and phenotyping

Whole blood or buccal swab samples were collected from privately-owned dogs with the owner’s consent mainly from Finland but also some cases and controls from France, Germany, Sweden and UK. The samples were recruited through breed clubs, scientific collaborators, veterinarians, and advertisement in various dog fancier forums. The samples were stored at the Dog DNA bank at the University of Helsinki along with the dogs’ health and pedigree information. The selection of suspected IE cases and controls was performed as described previously [11]. The suspected diagnosis of IE was based on a 10-page epilepsy questionnaire available in multiple languages (www.koirangeenit.fi) [15]. The healthy controls were not first or second degree relatives to the cases or to other controls. Genomic DNA was extracted from whole blood samples and buccal swabs using the Puregene DNA Purification Kit (Gentra Systems) and Chemagic Magnetic Separation Module I (MSM I) (Chemagen Biopolymer-Technologie AG, Baeswieler, Germany) according to the manufacturer’s instructions.

### Clinical examination

Altogether, 24 Kromfohrländers (17 cases, 7 controls), 12 Finnish Lapphunds (9 cases, 3 controls) and 12 Pyrenean Shepherds (10 cases, 2 controls) were clinically studied at the Referral Animal Neurology Hospital Aisti, Vantaa, Finland as described previously [7, 10]. The results of the clinical studies for Australian Shepherds have been reported previously [15]. Clinical studies were not performed for the other breeds.

### Genome-wide association analyses

GWA analyses were conducted for four breeds: Finnish Lapphunds, Kromfohrländers, Miniature Pinschers and Pyrenean Shepherds. The number of samples in each breed and genotyping platforms used are presented in Table 1. The samples were genotyped either with the Affymetrix Canine Genome 2.0 Array platinum set (Affymetrix, Santa Clara, CA, USA) with ~50,000 single-nucleotide polymorphism (SNP) markers or with the Illumina Canine HD Bead chips (Illumina, Inc., San Diego, CA, USA) for more than 173,000 SNPs across all canine chromosomes. The samples were genotyped at the Centre National de Génotypage (Paris, France) as part of the EU-funded LUPA project [16]. The genotype data was analysed with GenABEL v. 1.6–7 [17] in R v. 2.13.0. The Affymetrix GWA data was filtered for minor allele frequency > 0.05, genotype call rate > 0.75, individual call rate > 0.75 and Hardy-Weinberg equilibrium *p* > 0.0001. The Illumina GWA data was filtered for minor allele frequency > 0.05, genotype call rate > 0.95, individual call rate > 0.95 and Hardy-Weinberg equilibrium *p* > 0.00001. Multidimensional scaling plots were produced for each genotyped breed to identify outliers and population stratification. Identity-by-state values were calculated for each pairs of individuals to control for duplicate samples and relatedness. The GWA analyses were performed using mixed model approach in GenABEL [17, 18]. The estimation of polygenic model was performed using the polygenic option in GenABEL [19]. If the genomic inflation factor λ was >1, the association *p*-values were corrected for it. The quality control and association analyses were performed separately for each breed.

**Table 1:** Sample numbers, GWA platforms and sample origin

	GWA				ADAM23 SNP genotyping		
Breed	N cases	N controls	Sample origin^a^	Platform	N cases	N controls	Sample origin
Australian Shepherd	na	na	na	na	61	75	GER, FIN
Finnish Lapphund	40	97	FIN, SWE	Illumina 170 K	42	53	FIN, SWE
Irish Setter (red)	na	na	na	na	22	46	FIN, UK
Kromfohrländer	21	21	FIN	Affymetrix 50 K	46	29	FIN
Labrador Retriever	na	na	na	na	32	52	FIN, GER
Miniature Pinscher	15	15	FIN	Affymetrix 50 K	16	15	FIN
Pyrenean Shepherd	20	27	FIN,FRA	Illumina 170 K	18	23	FIN, FRA
Whippet	na	na	na	na	17	25	FIN, GER
Total	96	160			254	318	

*GWA* genome-wide association; na: not available, ^a^Samples are mainly collected in these countries, FIN: Finland; FRA: France; GER: Germany; SWE: Sweden, UK: United Kingdom

### ADAM23 haplotype analysis

To replicate the previous findings in CFA37, we estimated the required sample size using the Genetic Power Calculator [20]. The parameters for power calculation derived from our previous article [10]. The statistical power of our study materials were as follows: Australian Shepherds 0.69, Finnish Lapphunds 0.55, Irish Setters 0.38, Kromfohrländers 0.43, Labrador Retrievers 0.47, Miniature Pinschers 0.22, Pyrenean Shepherds 0.26 and Whippets 0.27. The statistical power in the whole replication material was 0.99.

Six variants (4 SNPs and 2 indels) composing an epilepsy risk haplotype according to our previous study [11] were selected for genotyping. The genotyping was performed using competitive allele-specific PCR (KASP) assays [21] (LGC, Herts, UK) and ABI PRISM 7900HT Sequence Detection System instrument (Applied Biosystems, Foster City, CA, USA) according to the protocols provided by the manufacturers. The total call rate was 0.94, but only 0.83 in the Irish Setters. The SNPs were in Hardy-Weinberg equilibrium (HWE) (*p* > 0.05) in the controls except in the Labrador Retrievers the HWE *p*-values were >0.01. The alleles and six-variant haplotypes were analysed for association using PLINK v. 1.07 [22]. Combined association analysis was performed using the Cochran-Mantel-Haenzel-test to control for the differences between breed clusters. The associations for homozygosity were calculated using the EpiTools package in R (https://medepi.com/epitools/). Per individual per haplotype missingness of < 0.5 was required in the haplotype analysis. The number of dogs included in this analysis is given in Table 2.

**Table 2:** Association results of epilepsy and the *ADAM23* risk haplotype^a^ in eight dog breeds

Breed N cases/controls	Risk haplo cases freq	Risk haplo control freq	*p*-value	OR (95% C.I.)	Risk haplo homozygosity cases freq	Risk haplo homozygosity controls freq	*p*-value	OR (95% C.I.)	Cases/controls without risk haplotype
Australian Shepherd 59/71	0.66	0.52	**0.03**	1.8 (1.1–3.0)	0.46	0.32	0.11	1.8 (0.9–3.6)	14%/28%
Finnish Lapphund 35/50	0.46	0.50	0.58	0.8 (0.5–1.6)	na	na	na	na	na
Irish Setter (red) 19/36	0.97	0.90	0.17	4.0 (0.5–33.7)	0.95	0.83	0.22	3.6 (0.4–32.4)	0%/3%
Kromfohrländer 46/29	0.88	0.69	**0.004**	3.2 (1.4–7.7)	0.83	0.45	**0.0008**	5.5 (1.9–16.8)	7%/7%
Labrador Retriever 29/50	0.59	0.33	**0.003**	2.9 (1.5–5.6)	0.34	0.16	0.06	2.8 (0.9–8.1)	17%/50%
Miniature Pinscher 16/15	0.88	0.7	0.05	3.3 (0.9–14.0)	0.75	0.53	0.21	2.5 (0.5–13.0)	0%/13%
Pyrenean Shepherd 17/23	0.65	0.43	0.06	2.3 (0.9–6.0)	0.47	0.13	**0.02**	5.5 (1.2–31.8)	18%/26%
Whippet 17/25	0.94	0.58	**0.0002**	11.3 (2.9–81.5)	0.89	0.36	**0.0005**	12.6 (2.7–102.3)	0%/20%

*P*-values <0.05 are bolded

*OR* odds ratio, *C.I*. confidence interval, *na* not analysed

^a^Haplotype *T*-*C*-*del*-*del*-*G*-*G* at 15,085,438 (BICF2S23030950), 15,106,446 (BICF2P1021781), 15,108,593, 15,108,802, 15,111,724 (BICF2P1131874) and 15,113,325 bp in *ADAM23* (~28 kb), respectively (CanFam3.1)

## Results

### Epilepsy phenotypes

The main characteristics of IE of the Finnish Lapphund, Irish Setter, Kromfohrländer, Labrador Retriever, Miniature Pinscher, Pyrenean Shepherd and Whippet cohorts were collected through detailed questionnaires, and are presented in Additional file 1: Table S1. IE in the seven breeds show typical onset at early adulthood, on average at the age of 2–4.5 years (range 4 months–8 years), and manifest with both focal and generalised seizures. A high variability in the seizure frequency is present in all breeds ranging from one reported episode in 2 years to several episodes per month. The characteristics of epilepsy of Australian Shepherds have been reported previously [15]. Besides questionnaire-based survey of the seizure history, clinical examinations were performed in selected dogs representing three breeds (Kromfohrländers, Finnish Lapphunds, Pyrenean Shepherds) to exclude possible external causes of seizures. None of the exams revealed abnormalities in the cases or controls, thereby supporting the diagnosis of IE or genetic epilepsy in those breeds.

### Genome wide association results

In order to identify loci underlying canine IE, GWA analyses were performed in altogether four dog breeds: Finnish Lapphunds, Kromfohrländers, Miniature Pinschers and Pyrenean Shepherds. No associations were identified in any of the studied cohorts (Additional file 2: Figure S1).

### Replication of the *ADAM23* association

Single-variant association analyses showed association (*p* < 0.05) in multiple breeds in *ADAM23* (Additional file 3: Table S2) with odds ratios ranging between 1.7 and 12.3 depending on the breed. The epilepsy-risk conferring alleles identified in our previous study were the major alleles in all the breeds, and they were more common among cases than controls in all breeds except for Finnish Lapphunds. A combined analysis of all breeds showed strong statistical significance (*p* = value 4.6e^−6^, OR 1.9, 95% CI 1.44–2.5 at 15,108,593 bp (genome build CanFam3.1). Combined analysis without Finnish Lapphunds showed the highest statistical significance of *p* = 2.5e^−8^ (OR 2.4, 95% CI 1.8–3.3) at 15,108,593 bp. Association analysis combining all associated breeds from our previous [11] and current studies yielded the highest statistical significance of *p* = 4.1e^−18^ (OR 2.6, 95% CI 2.1–3.2) at 15,108,593 bp.

The six-variant risk haplotype identified in our previous study [11] consisted of variants *T*-*C*-*del*-*del*-*G*-*G* at 15,085,438 (BICF2S23030950), 15,106,446 (BICF2P1021781), 15,108,593, 15,108,802, 15,111,724 (BICF2P1131874) and 15,113,325 bp in *ADAM23* (~28 kb), respectively (CanFam3.1). This haplotype showed association (*p* < 0.05) with IE in Australian Shepherds, Kromfohrländers, Labrador Retrievers, and Whippets (Table 2). The haplotype frequencies in Pyrenean Shepherds, Miniature Pinschers and Irish Setters showed a similar trend as in the other studied breeds, although the associations were not statistically significant. The *ADAM23* risk haplotype showed no evidence of association with IE in the Finnish Lapphund cohort.

## Discussion

In this study, we performed GWA analyses to four dog breeds with IE in order to identify novel breed-specific high-risk loci for the disease. As we previously reported that *ADAM23* is an important risk gene for canine IE [10, 11], we also investigated whether *ADAM23* plays a role in IE in an extended epilepsy collection, which consisted of eight additional dog breeds, and altogether 573 samples.

Most common human epilepsies are thought to have complex inheritance determined by multiple susceptibility loci with or without an environmental effect [23, 24]. Our study strongly suggests that the genetic background of common adult-onset IE does not follow simple Mendelian inheritance pattern in a number of dog breeds. The lack of association findings in the GWA studies performed in four breeds, Finnish Lapphund, Kromfohrländer, Miniature Pinscher and Pyrenean Shepherd, indicates inadequate statistical power, which points towards perhaps a more complex genetic background of the disease. There is ample evidence in canine genetics, that our sample cohorts were sufficient for the detection of loci harbouring causative genes for Mendelian disorders with high penetrance and without or with only few phenocopies [9]. Also, the SNP arrays may not efficiently capture all genetic variation in at least some of the studied breeds and this may be one of the reasons for the failure to find associations [11]. For example, the best associated marker BICF2P890779 in the first study [10] does not tag the locus in all breeds. The marker associated with IE in Belgian Shepherds, Whippets and Kromfohrländers, but not in Schipperke and Finnish Spitz. However, the new six-SNP haplotype, identified by targeted resequencing, shows association in all these breeds. Other non-*ADAM23*-linked risk loci possibly exist for canine IE, but their identification will require collection of extensive sample cohorts with rigorous phenotyping as well as denser marker maps or high-throughput sequencing.

In this study, we further confirmed *ADAM23* as a risk gene for canine IE. Although statistical power was limited in many breed cohorts, associations were identified in several breeds: Australian Shepherd, Kromfohrländer, Labrador Retriever, Pyrenean Shepherd, and Whippet. Finnish Lapphund was the only breed, which did not show any evidence of association despite adequate statistical power. This indicates either that *ADAM23* does not play a role in IE risk in this particular breed, or that the linkage disequilibrium pattern at the *ADAM23* locus differs in Finnish Lapphunds compared to the other studied breeds. Indeed, we may not yet have identified the causal variant in *ADAM23*, as the almost 90% GC content of its 5’ end has prevented us from investigating variation in *ADAM23* promoter region. The six variants composing the previously identified IE risk haplotype are located between exons 5 and 11 in *ADAM23*, at least 88 kb away from the 5’ end.

Thus far, *ADAM23* remains the only confirmed risk gene for the common IE in dogs, and we now have evidence for its role in IE in altogether 11 breeds. The identification of the causative variant at this locus will be included in our future experiments. In addition, we will investigate the function of ADAM23 in canine neurons in order to improve the understanding of the aetiology of canine IE. Our future experiments will also include whole-genome and whole-exome analyses of IE-affected dogs in order to identify novel genes for canine IE.

## Conclusions

Idiopathic epilepsy is the most common neurological disorder in dogs. Genetic cause for the disease has been suspected in many breeds based on high disease prevalence and pedigree analyses. However, only few disease genes have been identified to date. Our results further support the role of *ADAM23* in multiple dog breeds as a common risk gene for epilepsy with low penetrance. The lack of findings in the GWA analyses points towards inefficient capture of genetic variation by the current SNP arrays, causal variant(s) with low penetrance and possible phenocopies. Our results warrant studies on ADAM23 function and expression in canine neurons to improve the understanding of the molecular pathogenesis of common canine epilepsy.

## Additional files

Additional file 1: Table S1. Summary of the epilepsy questionnaires. (XLSX 13 kb)
Additional file 2: Figure S1. Genome-wide association study results. The multidimensional scaling plots (i), quantile-quantile plots (ii) and Manhattan plots (iii) are presented for Finnish Lapphunds (A), Kromfohrländers (B), Miniature Pinschers (C) and Pyrenean Shepherds (D). In the multidimensional scaling plots, red circles denote cases and blue controls. (TIF 1000 kb)
Additional file 3: Table S2. Allelic association results of the *ADAM23* variants. (XLSX 16 kb)
