# Supplementary figures and images for: ADAM23 is a common risk gene for canine idiopathic epilepsy
Source: BMC Genet. 2017 Jan 31;18:8. doi: 10.1186/s12863-017-0478-6 (PMC5282852; doi:10.1186/s12863-017-0478-6)

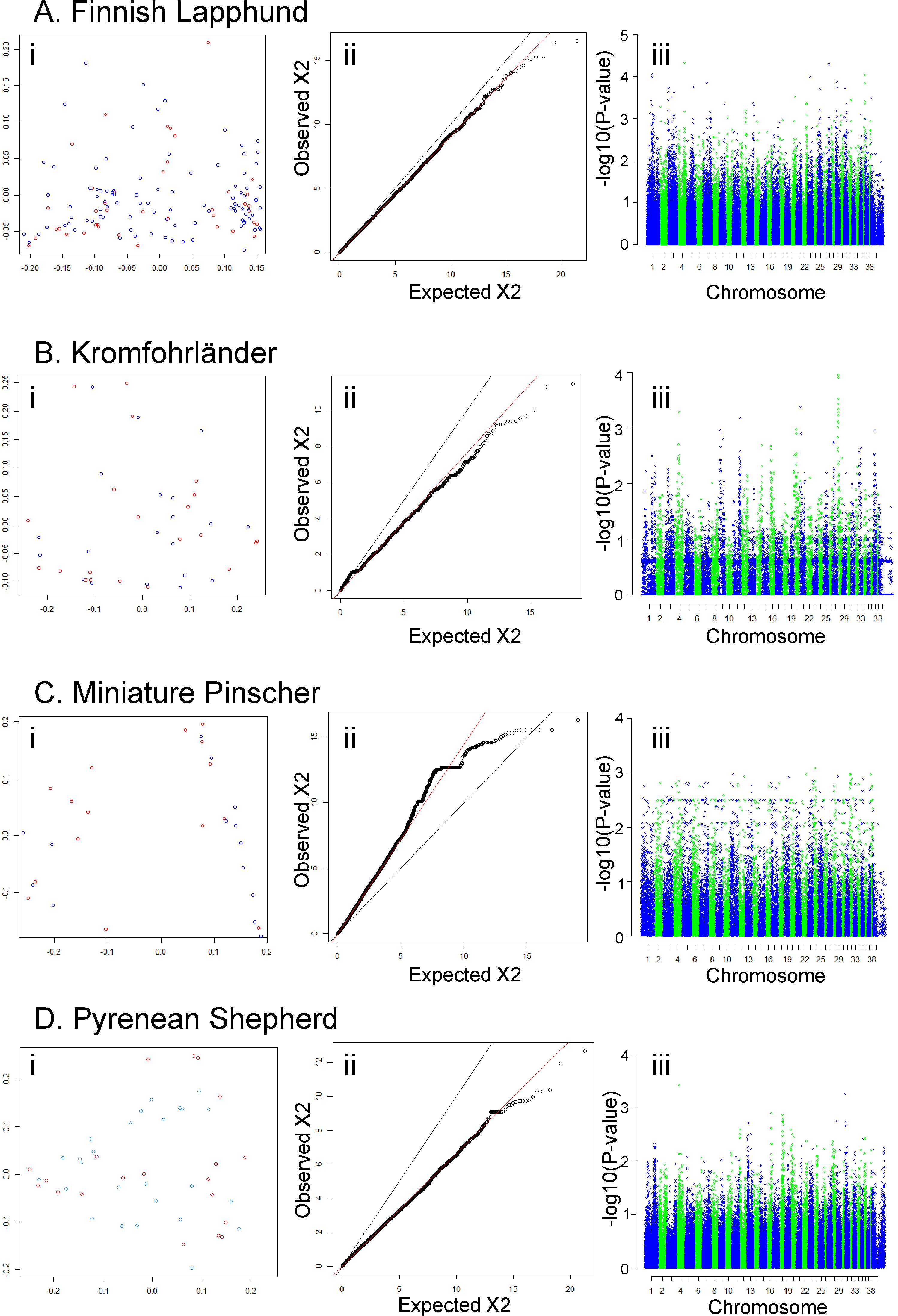

Supplement: Additional file 2: Figure S1. — Genome-wide association study results. The multidimensional scaling plots (i), quantile-quantile plots (ii) and Manhattan plots (iii) are presented for Finnish Lapphunds (A), Kromfohrländers (B), Miniature Pinschers (C) and Pyrenean Shepherds (D). In the multidimensional scaling plots, red circles denote cases and blue controls. (TIF 1000 kb) [file 12863_2017_478_MOESM2_ESM.tif]
